# Topological Defects Created by Gamma Rays in a Carbon Nanotube Bilayer

**DOI:** 10.3390/nano13030410

**Published:** 2023-01-19

**Authors:** Halina Grushevskaya, Andrey Timoshchenko, Ihor Lipnevich

**Affiliations:** Physics Department, Belarusian State University, 4 Nezalezhnasti Ave., 220030 Minsk, Belarus

**Keywords:** graphene, radiation hardness, pseudo-Majorana fermion, carbon nanotube

## Abstract

Graphene sheets are a highly radiation-resistant material for prospective nuclear applications and nanoscale defect engineering. However, the precise mechanism of graphene radiation hardness has remained elusive. In this paper, we study the origin and nature of defects induced by gamma radiation in a graphene rolled-up plane. In order to reduce the environmental influence on graphene and reveal the small effects of gamma rays, we have synthesized a novel graphene-based nanocomposite material containing a bilayer of highly aligned carbon nanotube assemblies that have been decorated by organometallic compounds and suspended on nanoporous Al${}_{2}$O${}_{3}$ membranes. The bilayer samples were irradiated by gamma rays from a ${}^{137}$Cs source with a fluence rate of the order of $10^{5}$ m${}^{-2}$s${}^{-1}$. The interaction between the samples and gamma quanta results in the appearance of three characteristic photon escape peaks in the radiation spectra. We explain the mechanism of interaction between the graphene sheets and gamma radiation using a pseudo-Majorana fermion graphene model, which is a quasi-relativistic N=3-flavor graphene model with a Majorana-like mass term. This model admits the existence of giant charge carrier currents that are sufficient to neutralize the impact of ionizing radiation. Experimental evidence is provided for the prediction that the 661.7-keV gamma quanta transfer enough energy to the electron subsystem of graphene to bring about the deconfinement of the bound pseudo-Majorana modes and involve C atoms in a vortical motion of the electron density flows in the graphene plane. We explain the radiation hardness of graphene by the topological non-triviality of the pseudo-Majorana fermion configurations comprising the graphene charge carriers.

## 1. Introduction

Applications of graphene-like materials are highly anticipated due to extreme mobility of their charge carriers [1,2,3]. Among them, the development of two-dimensional (2D) radiation-resistant materials and shielding nanostructured coatings are in great demand [4]. The radiation tolerance of multiwalled carbon nanotubes (CNTs) is demonstrated by the ability of carbon atoms to be displaced after gamma-ray exposure only in the vicinity of the graphene plane, as the radiation-induced structural rearrangement is restricted to chemical cross-links between the carbon atoms from the nanotube and the nearest carbon atoms from the environment [5,6]. Currently, there are two noticeable trends in the exploitation of the radiation hardness of graphene [7] and other 2D and plane-based materials [8]. On the one hand, as a promising tool for single atom manipulation, the radiation 2D damage provides a basis for nanoscale defect engineering. The individual radiation-induced defects in graphene have been used to obtain artificial ultraheavy atoms with collapsing atomic states. These artificial atoms are utilized as quantum devices called electrostatically confined p-n (n-p) graphene junctions (graphene quantum dots) to solve the problem of contacts in graphene nanoelectronics [9]. The 2D lattices of the nanodefects, which act as a molecular sieve, are needed, for example, for single-molecular DNA sequencing, obtaining ultrapure deionized water, or separating isotopes, as well as for supercapacitors (see [10,11,12] and the references therein). On the other hand, surprisingly, it appeared that the radiation-induced 2D defects could disappear [13]. This signifies that the 2D materials are, to a large extent, insensitive to ionizing radiation. Graphene is capable of self-healing after radiation damage. For example, monolayer graphene sheets may self-repair defects induced by the impact of 180-keV Xe${}^{40+}$, which shows that the graphene structure is perfectly self-healing [14]. Graphene holds the ability to self-heal, even in the presence of substitutional impurity atoms. When “jumping” through the graphene lattice at 60-keV electron irradiation, heteroatoms residing in a trigonal “*A*” (“*B*”) graphene sublattice, together with the closest neighboring carbon atoms residing in a trigonal “*B*” (“*A*”) graphene sublattice, may function as information bits “0” (“1”) in new-generation nanoscale memory arrays [15]. The properties of graphene provide the stimulus for developing novel materials for sensing fissile materials and for designing a new generation of devices for electronics used in spacecraft and nuclear facilities (graphene-based detectors for the detection of ionizing radiation) [16].

The understanding of the interactions between energetic particle beams and solids allows us to predict the behavior of three-dimensional (3D) systems under energetic particle beam irradiation, which occurs as a result of projectile–nuclei collisions, leading to the loss of the atom’s structural integrity and the formation of an ion and a charged vacancy. In contrast, vacancies induced by beta and ion radiation in graphene form neutral clusters [6,9,17]. The neutral atoms, which are knocked out of the graphene plane as a result of the exposure to high-energy (1 MeV–1 GeV) ions [5,18,19], electrons (see [17,19,20,21] and references therein), protons, and positrons (see [1,19] and references therein), cannot escape from the graphene plane to enter the deep bulk region. This demonstrates that the knock-on neutral carbon atoms reside in the graphene plane. The impressively ultra-high radiation hardness may be because the transversal electric charge transfer, which is caused by the impact of the ionizing radiation, is compensated for by the 2D charge transfer to the irradiation defects in the monolayer plane. It was observed experimentally [14] that this 2D charge flow neutralizes projectile ions. After the elastic scattering of relativistic ions on carbon atoms, the ultrafast transfer of slow (0–10 eV) electrons (see [22] and references therein) replenishes the ions’ electron shells within 1 fs, which prevents atom ionization and results in a few knock-on neutral carbon atoms (atoms displaced from their lattice sites by irradiation [23]) ending up outside the hexagonal lattice sites. To ensure the charge transfer to the projectile ions, the projectile particles should reside within the graphene plane during the transfer. Since the duration of the transfer is a few femtoseconds, a significant transfer of the charge and energy of the projectile to the nuclear and electronic graphene subsystems (σ- and π bond networks) is not observed. The giant graphene charge carrier flows must be generated because graphene needs a huge number of electrons to decelerate, even slow, highly charged ions. The giant current of the surface charge of the order of $10^{12}$ A/cm${}^{2}$ emerges to create the charge-carrier density equal to $3\times 10^{16}$ cm${}^{-2}$, which is necessary for the neutralization of the ion within five femtoseconds [14]. Such high values of the current density are not achievable in electrophysical experiments because the maximum currents measured experimentally in the monolayer graphene and single-walled carbon nanotubes (SWCNTs) or graphene nanoribbons are of the order of $1.18\times 10^{8}$ A/cm${}^{2}$ [24] or less than $10^{8}$ A/cm${}^{2}$ [25], respectively.

One of the mechanisms of 2D radiation damage is the 3D mechanism of a knock-on atom [26], which is based on a Lindhard atom screening effect [27], where incoming particles that do not interact with the electrons are elastically scattered by the crystal-plane atom potential in graphene or ultrathin crystals. Subsequently, the surface diffusion of the knock-on neutral C atoms proceeds very fast in the vacancy places (vacancy annealing) [28]. However, the existence of Coulomb interactions between the electrons from atomic clusters of the 2D material as a source of low-energy electrons contradicts the 3D mechanism of the knock-on atom. When the Coulomb-type 2D interactions have the greatest impact [29], attempts to explain defect production in graphene using the 3D mechanism are meaningless.

At present, the 2D mechanism of defect production, which is called interatomic Coulombic decay (ICD), is considered the prevalent mechanism of X-ray scattering in graphene [30,31] because the ICD is an efficient source of slow electrons. The ICD has been proposed as the prevalent mechanism of X-ray momentum relaxing in dimer molecules [32]. One of the two parts of the dimer is excited as a result of a Compton interaction with a projectile particle (photoionization), and then a photoelectron forms a repulsively bound pair with the other electron residing on the nearest lattice site (interatomic Coulombic electron capture) due to the Hubbard gap [33]. It was assumed in [34] that the ICD can mimic the explosive divergence of two repulsive bound electrons in an atomic cluster. An approach using a Hubbard-type Hamiltonian model (the Hartree approximation without taking into account the Fock exchange term) to describe the repulsive bound electron state was based on the quantum-field picture, which assumes that a cloud of virtual fermionic excitation pairs exists in the same lattice site (see [35,36] and references therein).

However, competing with the Hartree self-consistent field, the Fock exchange impedes the repulsion process (see [37] and references therein) and, correspondingly, weakens the correlations. Then, the probability of creating the repulsively bound pair state decreases, and, correspondingly, the transition to the Coulomb explosion state is weakened. This means that the neutralization current density has to be even higher. Moreover, according to [38], the ICD of 2D materials both with and without band gaps proceeds in the same way. Taking into account the above and the fact that ultrafast ICD proceeds at times comparable to the time scale of electronic motion (see [32] and references therein), the ICD theory is not applicable to the interaction of γ-rays, swift heavy ions, and slow highly charged ions with graphene.

Moreover, the decoupling of charge and heat currents is observed in graphene [39]. The breakdown of the Wiedermann–Franz law is due to the existence of a strongly interacting quasi-relativistic electron-hole plasma called a Dirac fluid. This decoupling is a sign of another mechanism based on the presence of vanishingly small graphene Fermi surface.

So, it is necessary to propose novel methods for predicting the state of outgoing particles from the graphene monolayer to implement the concept of defect engineering and advance new device designs for nuclear applications. However, the ability of neutral vacancies to be clustered and their relation to graphene’s ability to self-heal from radiation damage are still not fully understood. An explanation for the appearance of a large number of massless charge carriers in undoped irradiated graphene could provide a clue. However, there are no reasonable assumptions about the ways in which the energy of projectile particles is redistributed in graphene, making it difficult for the theory of interaction between graphene and radiation to predict the numbers and types of defects.

**Figure 1:**
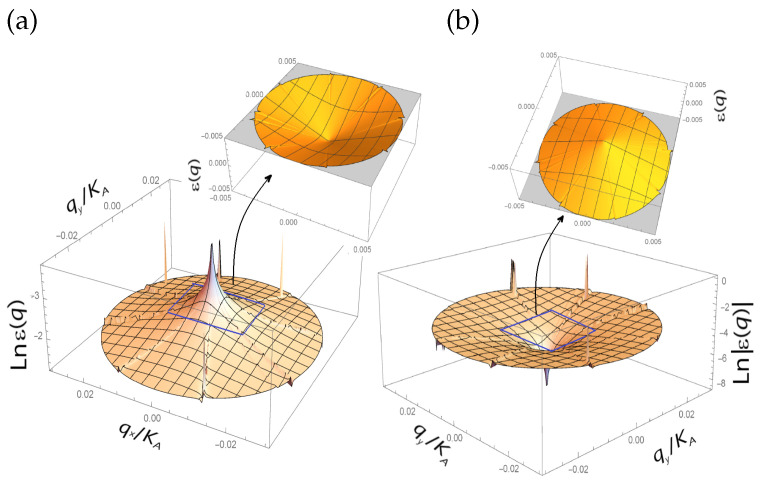
Electron (**a**) and hole bands (**b**) of graphene in the quasi-relativistic N=3-flavor fermion model with a pseudo-Majorana mass term.

The radiation-induced bound states are more likely to be midgap quasiparticle states, for example, Majorana quasiparticle states [40]. Formally, the density of graphene charge carriers can be infinite due to the zero energy of electron-hole pairs produced at the points where the graphene valence and conducting bands touch. These points are called the Dirac points, which reside in valleys $K,K^{\prime }$ of the graphene Brillouin zone. The charge carriers in a quasi-relativistic model of graphene are described by equations of a pseudo-Majorana type [41,42]. The pseudo-Majorana quasiparticles are topologically nontrivial. The topological vortical defects are subject to non-Abelian statistics. The electronic energy band structure of the quasi-relativistic N=3-flavor graphene model has been calculated with a Majorana-like mass term and is shown in Figure 1. The graphene bands are conical near the Dirac point at the momenta $q(q^{\prime })\to 0$, $q=\left|\vec{p}-\vec{K}\right|$ ($q^{\prime }=\left|\vec{p}\;{\;}^{\prime }-{\vec{K}}^{\prime }\right|$), where $\vec{p}\left(\vec{p}\;{\;}^{\prime }\right)$ is a momentum of an electron (hole). However, the bands flatten at large $q(q^{\prime })$.

Huge state densities called van Hove singularities (VHSs) exist in the graphene flat bands, where the VHSs are pushed to the Fermi level $E_{F}$ through high-level doping or structural deformation of the graphene under an environmental influence on the strongly correlated graphene electrons [43,44]. Surprisingly, the emerging instabilities are not affected by the vacancies [45]. The deviation of the graphene bands from the linear energy dispersion attributed to the valleys $K,K^{\prime }$ leads to an increase in the optical absorption at high energies [46]. This highly effective doping can be achieved, for example, through the Majorana transport channel, which is contributed by the scattering mechanism in strongly correlated systems involving, for example, quantum dots [47].

The stability of graphene under gamma-ray irradiation can be caused by interactions between the gamma quanta and pseudo-Majorana-type pairs of correlated graphene charge carriers, as sharp increases in the conductivity of rolled-up-graphene have been observed just after gamma-ray exposure at sufficiently low fluence [48,49]. When doping, the impact of the ions could colossally boost the conductivity of graphene compared to conventional doping, where the maximum number of charge carriers per one lattice site is of the order of $5.2\times 10^{-3}$ at a charge-carrier concentration of $3.4\times 10^{13}$ cm${}^{-2}$ [50].

Thus, the pseudo-Majorana quasiparticles, which comprise configurations of the Dirac fermion pair, can be candidates to play the role of repulsively bound states. Signatures of Majorana excitation were observed for graphene in [51]. The discovery of unconventional superconductivity in bilayer graphene also demonstrates the Mjorana-like behavior of the fermionic excitations in graphene [52]. The interaction between the gamma-ray beam and the super-dense flux of the charge carriers entering the pseudo-Majorana configurations can invoke a Compton effect in the graphene plane. However, such processes have not yet been studied.

In this paper, we use the pseudo-Majorana graphene model to explain how ionizing radiation interacts with nanostructures based on graphene.

The electronic structure of a graphene monolayer is strongly influenced by the environment. Since both the environment and graphene sample are exposed to radiation and the experimental conditions and samples are not identical, it is very difficult to detect the weak contribution of radiation damage to the change in the intensity of graphene Raman bands due to the significant influence of the environment [53]. The environmental influence is revealed by scanning tunneling microscopes and transmission electron microscopy (TEM) images of carbon nanotubes, which show a mismatch between the thickness and cross-section of the rolled-up graphene sheets of single-walled carbon nanotubes [54,55]. To avoid this, it is necessary to utilize a large number of identical graphene samples suspended on nanoporous substrates in order to prevent the doping of graphene from the side of the substrate, which is polarized and electrified due to the impact of beams of high-energy particles on a target. Currently available technologies for fabricating carbon fibers and yarns do not produce satisfactory ordering, making it difficult to obtain visible and reliable insights into the impact of gamma rays on graphene charge carriers through Raman light scattering in comparison to the contribution of the impurity states [17]. Therefore, the study of the 2D damage produced by gamma-ray irradiation required the use of practically defect-free graphene-based nanomaterials. However, their development is a challenge [56]. The mechanisms of interaction between graphene charge carriers and the 3D electromagnetic environment remain elusive due to the lack of such materials.

In this work, to eliminate environmental influences, we fabricate bilayers of highly aligned carbon nanotube assemblies that are decorated by organometallic compounds and suspended on nanoporous Al${}_{2}$O${}_{3}$ membranes. We study the scattering of gamma radiation on rolled-up graphene sheets of carbon nanotubes from the aligned assemblies and show that fluxes of “vortex–antivortex” pairs are produced by gamma quanta in the graphene rolled-up planes of the carbon nanotubes.

The goal of the paper is to reveal and explain the mechanisms responsible for producing high-energy free vortex–antivortex pairs in the electron density of graphene through the breaking of hexagonal symmetry upon impact with a low-intensity gamma beam. To achieve this, we use the pseudo-Majorana graphene model. We propose that the radiation hardness of graphene stems from the production of topological defects.

## 2. Materials and Methods

### 2.1. Reagents

Few-walled CNTs (FWCNTs) with a diameter of 2.5 nm and a length of ∼5–10 μm were produced using the chemical vapor deposition method (CVD method). Then, the raw FWCNTs, which were purchased from Fibermax (Greece), were covalently modified by carboxyl groups and non-covalently functionalized by stearic acid molecules. Then, the carboxylated and stearic-acid-functionalized FWCNTs were decorated by nanocyclic complexes of Ce and/or high-spin octahedral Fe with ligands as a conducting oligomer 2,5-di(2-thienyl)-1H-pyrrole (2,5-di-(2-thienyl)-pyrrole) in the following manner [57]. As a preliminary, an alkyl hydrocarbon chain C${}_{16}$H${}_{33}$ was linked chemically to the oligomer. The chemical formula for the oligomer labeled by the abbreviation “H-DTP” (H-dithienylpyrrole) is 3-hexadecyl-2,5-di(thiophen-2-yl)-1H-pyrrole. Here, the part of H-DTP without a hydrogen atom is denoted as “DTP”.

### 2.2. Methods

#### 2.2.1. Exposure to Radiation

A standard low-intensive source of ionizing radiation (IRS) ${}^{137}$Cs (CsJ) was used. A low-intensive beta-particle beam from the IRS was attenuated by a thin-film aluminum shield. The radiation source has the form of a drop with an average diameter of about d=1.5 mm. An absolute IRS activity $A_{0}$ equal to 124.4 kBq dated 1 April 1990 was quoted to about 1% precision. Correspondingly, the activity at the measurement moment (t=31 years) is

(1) $$A_{t}=A_{0}\exp\left(-t\ln2/T_{1/2}\right)=A_{0}/2.$$

**Figure 2:**
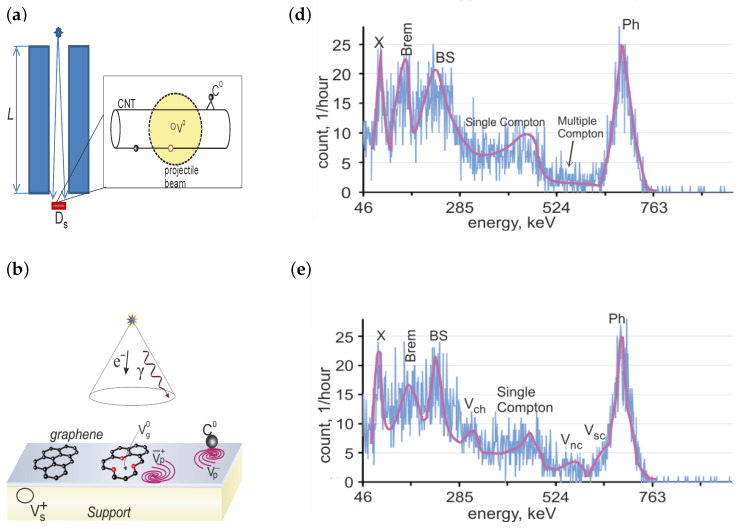

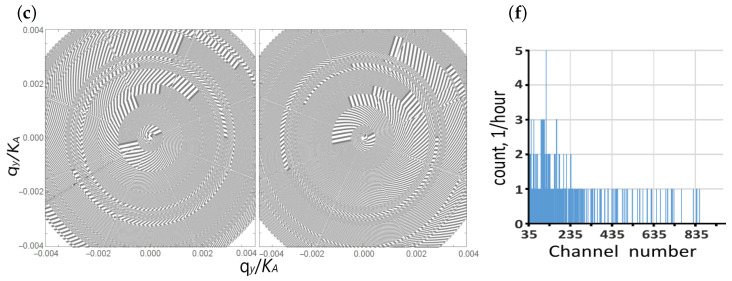
(**a**) Schematic of irradiation of a sample (underneath the collimator of length *L*) of diameter $D_{s}$. Inset: radiation-induced CNT defects as neutral graphene vacancies $V_{g}^{0}$ with knock-on neutral atoms C${}^{0}$. (**b**) Schematic of the ionization radiation impact. The knock-on C atoms are fixed on a nanotube wall (graphene rolled-up monolayer) by irradiation defect vortical pairs (pseudo-Majorana fermions), ${\bar{V}}_{p}^{+}$−$V_{p}^{-}$, schematically imaged in (**b**). Confining the vortices $V_{p}^{-}$ and antivortices ${\bar{V}}_{p}^{+}$ from the pairs near the points *K* and $K^{\prime }$ of the graphene Brillouin zone, respectively, the hexagonal symmetry binds the pseudo-Majorana quasiparticles. Each pair of bound pseudo-Majorana fermions produces a very high number (avalanche) of electrons and holes. The gamma and beta rays are indicated by γ and $e^{-}$, respectively. (**c**) The vortex texture as contour plots of the graphene electron (left) and hole (right) bands shown in Figure 1. The radiation spectra $R_{Cs}$ (**d**) and $R_{Cs/CNT}$ (**e**) for the IRS ${}^{137}$Cs photon beam incoming through the collimator without and with the bilayer, respectively, and scattered on the detector crystal. The backscatter peak, the photopeak, the characteristic X-ray peak, and the contribution from the bremsstrahlung are labeled as “BS”, “Ph”, “X”, and “Brem”, respectively; the bilayer photon escape peaks corresponding to the graphene −γ-ray interaction producing the pseudo-Majorana chiral, semichiral, and nonchiral fermions are labeled as “$V_{ch}$”, “$V_{sc}$”, and “$V_{nc}$”, respectively. The single Compton continuum and area of multiple Compton scattering are labeled as “Single Compton” and “Multiple Compton”, respectively. The radiation background has been subtracted from the radiation spectra. (**f**) The radiation background.

Here, $T_{1/2}$ is the half-life time of radioactive decay, $T_{1/2}=30.2$ years [58]. A sample with a diameter $D_{s}=4$ mm was exposed through a lead collimator of a 5 mm diameter and an *L* = 25, 50 mm length. The scheme of irradiation is shown in Figure 2a,b,c. The IRS was placed above the collimator. At the ratio d/L=0.03, 0.06, the IRS can be considered a point source. A gamma-radiation fluence rate in the irradiated sample can be estimated as

(2) $$\phi_{0}=\frac{kA_{t}}{4L^{2}}\frac{\pi D_{s}^{2}}{4L^{2}}$$

where *k* is the percentage of the emitted photons per one decay (quantum yield), $\frac{\pi D_{s}^{2}}{4L^{2}}$ is the solid angle under which the irradiated sample is viewed from the point IRS. For the IRS ${}^{137}$Cs, k=0.851. Therefore, $\phi_{0}=8.5\times 10^{3}$ m${}^{-2}$s${}^{-1}$ at d/L=0.03 and the increasing factor is $2^{4}$ at d/L=0.06. The number of gamma quanta coincides with the number of 0.512 MeV electrons emitted by ${}^{137}$Cs atoms in the decay process. Since the percentage of 1.174 MeV electrons is 5.3 %, the fluence of the beta-quanta with the energy 1.174 MeV is less than 485 m${}^{-2}$s${}^{-1}$ at d/L=0.03. The carbon nanotube bilayers were exposed to radiation for 1 or 3 h at d/L=0.03 and 86 min at d/L=0.06. We registered approximately 9200 events at d/L=0.06. The insulating glass-ceramic support becomes conductive upon exposure to β-radiation [59]. However, since in our case the intensity of the beta rays is very small due to the aluminum shield, the doping of the carbon nanotube bilayer is negligible.

#### 2.2.2. Radiation Spectroscopy

An analysis of the transmitted gamma rays was performed using the lab-quality radiation spectrometric equipment in the “Nuclear Physics” (BSU, Minsk, Belarus) training laboratory. A thallium-activated sodium iodide scintillation crystal NaI(Tl) (diameter of 25 mm, height of 40 mm) was utilized as a detector crystal. The technical characteristics of the radiation spectrometer were as follows. The photoelectric-multiplier (PEM) supply voltage *U* changed from 100 to 1000 V; after 20 min of warming, the voltage instability did not exceed 0.05% for 5 h of continuous apparatus operation; the admissible current of the PEM power supply was not less than 5 mA; the input resistance of the main amplifier was 15 kOm; the amplifier gain changed from 1 to 100 smoothly or stepwise; the transformable signal range was 0 to 10 V; the signal polarity was arbitrary (it was defined programmatically in the amplifier gain settings); the signal-front duration was no more than 0.3 μs; the maximal signal duration was 20 μs; the time for the data conversion and transmission to a computer accounting for the blocking scheme was 30 μs; the number of channels (a maximal pulse height) was 1024; the differential nonlinearity was no higher than ±1%; the integral nonlinearity (transducer characteristic error) was no higher than 0.1%; the displacement of the full-energy position was no more than 1% for the measurements of the 661.7-keV ${}^{137}$Cs gamma ray at load changes of a factor of 10.

The number $N_{event}$ of gamma quanta scattered in the detector crystal has been calculated by the summation of all numbers, $n_{i}$, $i=N_{d}+1,N_{d}+2,\ldots ,N_{u}$, of the pulse counts for the high-level channels (pulse heights) at U=650 V. The region (the numbers) of the low-level channels is 1 to $N_{d}=34$ and the region of the high-level channels is up to $N_{u}=1000$. The background scattering is practically absent in the high-energy channels (see Figure 2f). The number of 661.7 keV gamma photons recorded during the photoionization measurements is given by the area of the radiation peak. A linear conversion of the pulse height (channel number) $N_{c}$ into the energy *E*: $N_{c}\to E/a$ was performed with non-zero offset *b* as

(3) $$E_{0}=aN_{0}+b;\;E_{k}=aN_{k}+b$$

for the ${}^{137}$Cs gamma ray that deposited the energy $E_{0}=$661.7 keV in the photopeak channel $N_{0}=550$ and for the escaping gamma ray, which was a single Compton scattered at the angle 180${}^{\circ }$ and deposited the energy $E_{k}=E_{0}\left(1-\frac{1}{1+2E_{0}/\left(m_{e}c^{2}\right)}\right)=477.37$ keV in the detector channel $N_{k}=396$. Here, $m_{e}$ and *c* are the electron mass and the speed of light, respectively.

#### 2.2.3. Response Functions and Photoelectron Statistics

The response functions of the detector shown in Figure 2d,e have narrow peaks in the photoelectric absorption (full-energy peak, “photopeak”) and a characteristic X-ray at the highest and lowest pulse heights. The characteristic X-ray photons are emitted by free electrons filling non-occupied electron *K*-shells in the atoms of the lead collimator. The photopeak appears at the energy of the original ${}^{137}$Cs gamma-ray photon. Compton scattering gives rise to a single Compton continuum of energies and multiple Compton scattering events in the spectrum of the source gamma radiation. Multiple Compton scattering occurs in the large detector crystal. A peak caused by the bremsstrahlung generated due to stopping the beta particles by the IRS shield material was also observed. A peak in the vicinity of 230 keV is the backscatter peak caused by photons scattered at large angles in materials immediately surrounding the scintillator crystal [60].

The normalized full-energy peak of the ${}^{137}$Cs radiation spectrum without the background can be considered a probability distribution of the energy per gamma quantum scattered by an atom of the detector crystal. The fluctuations in the particle number resulted in peak broadening estimated by its variance σ. Let us assume the Poisson distribution of the relative number of counts per channel within the photoabsorbtion peak. Then, our calculation of the variance σ yields

(4) $$\sigma \equiv \sqrt{\langle k\rangle }∼\sqrt{550}\approx 23,$$

where 〈k〉 is the mean of the photoelectric peak height. At this variance value, the fidelity of the detection of the gamma-quantum contribution to the photoeffect recorded in the channels from 525 to 640 was equal to 5σ and is, therefore, confident (the probability of the gamma-quantum detection is more than 0.997) [61]. Thus, the setup tolerance for the measurements was less than δ=0.3%. Utilizing the Poisson distribution, the relative error for the estimation of the secondary photoelectron energy was less than $\delta^{ph}=\sigma /\langle k\rangle =0.043$. Let us assume that the measurements of the gamma-quanta fluxes $N_{Cs/CNT}$ and $N_{Cs}$ of the ${}^{137}$Cs with and without the absorber, respectively, were independent. Then, the estimation error for the difference, $N_{Cs}-N_{Cs/CNT}$, between the counts $N_{Cs}$ and $N_{Cs/CNT}$ is defined by the probability $p_{ab}$ of the detection of the $N_{Cs/CNT}$ and $N_{Cs}$ as

(5) $$p_{ab}=p_{a}p_{b}\approx {\left(1-\delta \right)}^{2}={\left(1-0.003\right)}^{2}=0.994.$$

#### 2.2.4. The Langmuir–Blodgett Technique and Ultrathin Absorber Materials

Preliminary, inverse micelles of stearic acid with the FWCNTs inside were obtained by mixing the stearic acid and FWCNTs in hexane by ultrasound treatment. Then, two or three carbon nanotube monolayers fabricated from these micellar FWCNTs using the LB technique were deposited on a Si support or suspended on 10 nm pores in anodic aluminum oxide (AOA). The highly ordered aligned carbon nanotube LB assemblies decorated with a 5-monolayer film of nanocyclic organometallic complexes, Fe(II)DTP, were deposited on the Si surface or suspended on an interdigital structure of aluminum electrodes, on the surface of which, the nanoporous AOA layer was previously formed as an insulator coating. The Fe(II)DTP film was fabricated using the LB technique. The interdigital electrode structure was deposited on glass-ceramic support such as pyroceramics [62].

#### 2.2.5. Structural and Diffraction Methods

Microdiffraction patterns and TEM images were obtained using a transmission electron microscope JEM-100CX (JEOL, Japan) at an accelerating voltage of 100 kV. The samples were previously deposited on a copper grid with a formvar polymer coating or 19 nm porous AOA membranes from the direction of the AOA barrier layer.

#### 2.2.6. Raman Spectroscopy

Spectral studies in the visible range were carried out using a confocal micro-Raman spectrometer Nanofinder HE (“LOTIS-TII”, Tokyo, Japan–Belarus) with lasers operating at wavelengths of 355 (external laser), 473 (diode pumped solid state (DPSS) laser), and 532 (DPSS laser) nm, with power in the range of 0.0001 to 20 mW. The spectra were recorded in back-scattering geometry under a ×50 objective at room temperature. Nanoporous AOA membranes with a pore diameter of 10 nm and Si supports were utilized in the Raman spectroscopic studies.

## 3. Physicochemical and Structural Characterizations of Aligned CNT LB Assemblies

**Figure 3:**
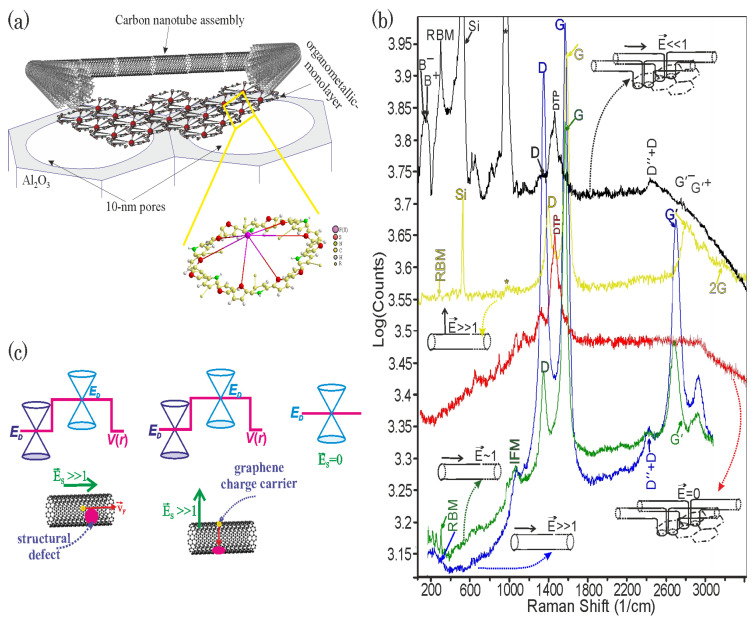
(**a**) Aligned CNT assembly within a bilayer suspended on AOA nanopores on which the organometallic compound monolayers were deposited preliminarily. Inset: structure of the organometallic nanocyclic complex Fe(II)DTP. (**b**) The Raman spectra for CNT two- and three-monolayer LB films deposited on different supports: yellow for the three-monolayer CNT film on Si, whose surface was preliminarily modified by a drop of the H-DTP solution in hexane; black and red for the LB films consisting of five Fe(II)DTP monolayers and two CNT monolayers deposited on Si (the black curve) and on the 10 nm porous AOA membrane (the red curve), whose surfaces were preliminarily modified by a drop of the H-DTP solution in hexane; green for the two-monolayer CNT film deposited on the 10 nm porous AOA, whose surface was preliminarily hydrophobized by the stearic acid; blue for the two-monolayer CNT film deposited on the 10 nm porous AOA, whose surface was preliminarily hydrophilized by the FWCNT solution in hexane. The Raman spectra were recorded at laser excitation wavelengths of 355 (the yellow line), 473 (the green and blue lines), and 532 (the black and red lines) nm; the following laser powers and collection times were used for the specimen excitation: 0.6 mW and 10 s (the red line), 2 mW and 30 s (the yellow line), 5.76 mW and 40 s (the green and blue lines), 14.4 mW and 10 s (the black line). Symbols “Si” and “*” denote the vibrational modes of Si at 519.57 cm${}^{-1}$ and the laser mode at a frequency of 980 cm${}^{-1}$, respectively. (**c**) Mechanisms of action of electrical fields, ${\vec{E}}_{s}$, from the CNT environment. The energy band, $E_{D}$, touching at the Dirac point in a graphene patch shifts under the action of ${\vec{E}}_{s}$ with and without the formation of resonances as a result of Klein tunneling at oblique (c, left) and transversal (c, middle) scattering of graphene charge carriers, $q_{G}$, respectively, on the potential barriers $V(\vec{r})$ of the structural graphene-plane defects induced by the dipole polarization and electrification of the support. The energy of the Dirac touching shift is lacking at the zero field ${\vec{E}}_{s}$ (c, right). The charge carriers move at the Fermi velocity, $v_{F}$.

The nanostructured composite material is shown schematically in Figure 3a. The Raman spectra of the ultrathin Fe(II)DTP LB film are indicated in Figure 3b, by black and red curves. The Raman scattering of light in the metal-containing LB film is resonantly enhanced on eigenfrequencies of the molecular dithienylpyrrole group by plasmon oscillations of the surface charge density of the Si support or the carbon nanotubes.

**Figure 4:**
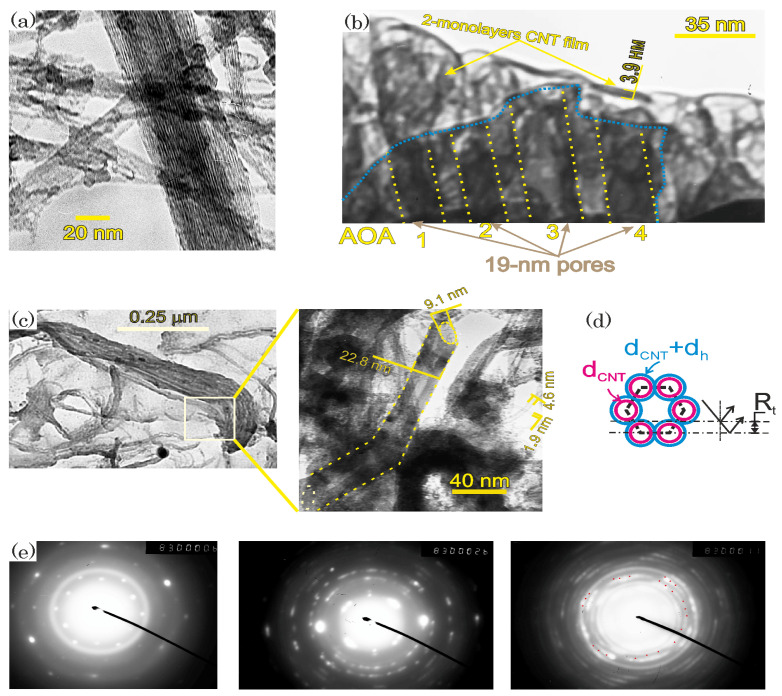
(**a**) TEM image of original carboxylated few-walled CNTs deposited on a copper grid at 270,000× magnification. (**b**–**e**) TEM images (**b**,**c**) and microdiffraction patterns (**e**) of two- and three-monolayer CNT LB films and a scheme of the diffraction (**d**). The magnifications are 140,000 (**b**), 19,000 (**c**, left), and 190,000 (**c**, right) times the original size. The CNT bilayers are arranged on the following supports previously covered by five (**b**,**d**, left and middle) or three (**c**) Fe(II)DTP monolayers: a fresh cleavage of AOA with a pore diameter of 19 nm (**b**) and copper grids (**c**,**d**). The three-monolayer CNT film, whose diffraction pattern is presented in Figure d, right, was deposited on the grid without the Fe(II)DTP covering. The dashed yellow lines provide a guide to the eye for the 19 nm AOA pores in Figure b, and the blue curved line indicates the CNT bilayer edge (**b**). The elliptic 9.1 nm cross-sections of the thick CNT assembly are indicated by yellow ovals (**c**). The inset marked by yellow lines in (**c**), shows the thick and thin CNT assemblies with apparent diameters of 22.8 and 4.6 nm, respectively, and the CNT with an apparent diameter of 1.9 nm.

The TEM images of the original carboxylated FWCNTs are shown in Figure 4a. The TEM images and Raman spectra of the Fe(II)DTP-decorated and non-decorated CNTs from the two- and three-monolayer LB films covering different types of support are shown in Figure 4b–c and Figure 3b. The Raman spectra of the CNTs indicate that a characteristic radial breathing mode (RBM) is in the spectra at values of 297.96 (blue spectrum at 473 nm laser excitation), 297.7 (yellow spectrum at 355 nm laser excitation ), 304 (green spectrum at 473 nm laser excitation), and 300.895 cm${}^{-1}$ (black spectrum at 532 nm laser excitation) for the non-decorated bilayer deposited on the 10 nm AOA nanopores, whose surfaces were preliminarily hydrophilized by the FWCNT solution in hexane, for the three-monolayer non-decorated CNT LB film deposited on Si, whose surface was preliminarily modified by a drop of the H-DTP solution in hexane; for the non-decorated CNT bilayer deposited on the nanoporous AOA, whose surface was preliminarily hydrophobized by the stearic acid, and for the decorated CNT bilayer on the Si surface modified by a drop of the H-DTP solution in hexane, respectively, (see Figure 3b). The values of the RBM frequency, $\omega_{RBM}$, depend on the single-walled CNT (SWCNT) diameter $d_{CNT}$ and a coefficient $C_{env}$ quantifying the environmental effect according to the following formula [63]:

(6) $$\omega_{RBM}=\frac{227}{d_{CNT}}\sqrt{1+C_{env}d_{CNT}^{2}}.$$

Let us estimate $C_{env}$ approximately. The coefficient is approximately equal to zero in the case of the non-decorated CNT bilayer suspended on the hydrophilized AOA nanopores when interaction with the substrate is excluded. Then, the CNT diameter is estimated to be of the order of 0.761 nm for the RBM of the order of 298 cm${}^{-1}$ at zero value of the $C_{env}$. CNTs with such diameters are single-walled. Using the following formula [64]:

(7) $$d_{CNT}=\frac{0.246}{\pi }\sqrt{\left(n^{2}+nm+m^{2}\right)}$$

one obtains the index (n,m)=(7,4) for the SWCNTs. This is a sign that the CNTs are a metal type because mod[(n−m),3]=0.

Split vibrational bands are observed in the Raman spectrum of the decorated CNT bilayer (black curve in Figure 3b). The characteristic frequencies in the spectrum are 136.047 (B${}^{-}$), 152.133 (B${}^{+}$), 300.895 (RBM), 1371.02 (*D*), 2443.19 ($D^{\prime \prime }+D$), 2747.14 (${G\prime }^{-}$, $2D^{-}$), and 2776.64 (${G\prime }^{+}$, $2D^{+}$). The higher frequency of the bending vibrational mode (B${}^{+}$) corresponds to the oscillations of the nucleus bending in the monolayer plane, whereas the lower frequency (B${}^{-}$) corresponds to the out-of-plane bending oscillations. A model of the bilayer structure is shown in the upper-right corner of Figure 3b. The split $G^{\prime }$ (2D) band (two-phonon Raman line with double the frequency of the defect-activated (D) peak) in the Raman spectrum of the decorated CNT bilayer on Si indicates the vibrations ${G\prime }^{+}$ and ${G\prime }^{-}$ of the twisted CNTs across and along the twist, respectively [55]. The environmental coefficient $C_{env}$ for the decorated CNT bilayer on Si is 0.0303 and is smaller than that of the non-decorated CNT bilayer on the hydrophobized nanoporous surface due to the lack of interactions in the direction orthogonal to the CNT axis.

A dielectric polarization of the support or a charge transfer may be induced under the applied electromagnetic field. The light-induced charge transfer leads to the electrization of the support surface. Electrical fields ${\vec{E}}_{s}$ are created by the support dipole polarization and electrification impact on the graphene plane. The types of impacts are schematically presented in Figure 3c. When breaking a graphene σ-bond network, the action of the electrical fields leads to the Dirac band touchings being shifted relative to each other for different graphene patches, forming potential barriers V(r) (see Figure 3c). The Dirac point shift is revealed through the characteristic “hybridization” *G* peak in the Raman spectra of the vibration modes for the carbon nuclei oscillating in the potential V(r) (see Figure 3b). The shift means that the graphene patches are doped. The currents of the massless pseudo-Dirac excitations of the $\pi (p_{z})$ electron density appear to neutralize the charged graphene defects. When the massless graphene charge carriers under the action of the support dipole polarization field, ${\vec{E}}_{s}$, are obliquely incident on the potential barrier V(r) of the graphene-plane defects the resonant states are produced as a result of Klein tunneling (see Figure 3c, left). The resonant states break the π-bonds. The carbon nuclei oscillate within the potential of the resonant states. The defects of the graphene π-bond network are revealed through the characteristic D peak in the graphene Raman spectra shown in Figure 3b. Since the potential barrier is completely transparent to the normally incident massless pseudo-Dirac particles, the intensity of the *D* peak decreases when the projection of the support electric field (namely, the spherically symmetrical defect in the support) onto the graphene plane decreases (see Figure 3c, middle). According to the Raman spectra recorded at laser excitation wavelengths of 355, 473, and 532 nm (see Figure 3b), the intensity, $I_{D}$, of the Raman *D* peak and/or ratio, $I_{D}/I_{G}$, of the $I_{D}$ to the Raman *G*-peak intensity, $I_{G}$, is the highest for the support surface dipole polarization when the resonances are created under the action of the support electrical field directed obliquely to the graphene plane. The ratio $I_{D}/I_{G}$ and intensity of the *D* peak decrease when the charge transfer occurs under the action of ultraviolet radiation because free electric charges and electrically charged impurity centers are created in the support and the electrical field of the charged centers is directed orthogonally to the graphene walls of the carbon nanotubes (see the Raman spectrum in yellow in Figure 3b). The Raman *D* and *G* peaks of the Raman spectra of the Fe(II)DTP-decorated bilayer become weakly intense (see the Raman spectrum in black in Figure 3b) and disappear for the bilayer suspended on the AOA nanopores (see the Raman spectrum in red in Figure 3b). This demonstrates that the effect of the electric fields from the sides of the supports is either attenuated owing to the remoteness of the nanotubes from the Si surface or disappears for the bilayer suspended on AOA nanopores. Correspondingly, the CNT bilayer deposited on the surface of the 5-monolayer Fe(II)DTP LB-film is more ordered and contains fewer defects.

The CNT bilayer and three-monolayer CNT LB films would produce two related microdiffraction spots corresponding to the SWCNTs of opposite chirality (whose axes are in opposite directions), or the diffraction spots associated with a range of CNT chiral angles would form arcs. The arrangement of the microdiffraction reflections shown in Figure 4e indicates only one diffraction spot for one monolayer of the CNT LB films. This signifies that monodispersed crystals are formed from SWCNTs with one CNT chiral angle. The two primary directions indicated by the hexagonal microdiffraction patterns correspond to the so-called tube-radius reflection $\left(R_{t}\right)$ of 0.512 nm in size and, orthogonal to this, a spacing of 0.236 nm corresponding to the spacing of the graphite hexagons (0.24 nm). According to the diffraction scheme shown in Figure 4d for the arrangement of the monolayer CNT that ends on a rhombic lattice, the tube-radius reflection, which is equal to half the distance between the nearest crystal planes passing through the centers of the CNT cross-sections, is connected to the nearest-neighbor carbon–carbon distance ($2d_{h}$) between atoms belonging to adjacent tubes by the following formula:

(8) $$R_{t}=\frac{1}{2}\left(d_{CNT}+2d_{h}\right)\cos\left(\pi /3\right)$$

where π/3 is the angle in the unit cell of the rhombic lattice. Using Formula (8) for the metallic SWCNTs of a 0.761 nm diameter, one obtains that $2d_{h}$ is equal to 0.42 nm. For comparison, the 0.34 nm distance between the nearest carbon atomic layers in graphite is less than that of the CNT end arrays due to stretching CNT bodies.

The effects of the structural gamma-radiation-induced damage of the nanotube monolayers are weak. Therefore, it is necessary to exclude the influence of the dipole polarization and electrization of the irradiated support. The lack of a Raman *D* peak (the red curve in Figure 3b) and the perfect alignment of the decorated CNTs for the bilayer suspended on the 10 nm diameter pores indicate that the aligned CNT assemblies and, correspondingly, the graphene walls of CNT, are free from the support-induced structural defects. Since the LB bilayers of the decorated carbon nanotubes suspended on the nanopores possess a defect-free crystal structure, they are perfect for detecting the small effects of gamma-ray exposure.

The gamma radiation experiments were conducted using only perfect graphene rolled-up planes.

## 4. Graphene Interaction with Photons: Model

### 4.1. Pseudo-Majorana Fermion Graphene Model

The band structure of the quasi-relativistic graphene model with the pseudo-Majorana fermions forming Dirac configurations of charge carriers hosts vortex and antivortex structures, whose cores are in the graphene valleys $\vec{K}$ and ${\vec{K}}^{\prime }$ of the Brillouin zone, respectively (see Figure 2c). Touching in the Dirac points $K,K^{\prime }$, the conic-like valence and conduction bands of graphene become more flattened at high momenta $\vec{q}=\vec{p}-\vec{K}$ ($\vec{q}\;{\;}^{\prime }=\vec{p}\;{\;}^{\prime }-{\vec{K}}^{\prime }$) of the graphene charge carriers [65]. This means that the Fermi velocity $v_{F}$ drastically decreases to very small values at large *q*. Since the eight subreplicas of the graphene energy band near the Dirac point are degenerated into the eightfold conical band (see Figure 1), the pseudo-Majorana fermions revealed through the eightfold vortices in the graphene Brillouin zone shown in Figure 2c are confined by hexagonal symmetry. In the confinement state, the pseudo-Majorana fermions are bound with the created electron-hole pairs.

The Zak phase of the graphene charge carriers entering the pseudo-Majorana configuration is non-zero. The charge carriers, whose non-Abelian Zak phase multiples of π/6 constitute the cyclic group $Z_{12}$, are confined near the Dirac point. The π/6 rotation is equivalent to a π/2 rotation due to the hexagonal symmetry of graphene and, correspondingly, the electron and hole configurations in the momentum space are orthogonal to each other. This demonstrates the metallicity of the zigzag edges and zigzag configurations and the semi-conductivity of the armchair edges and armchair configurations transversal to the zigzag configuration in the graphene plane. All $\pi (p_{z})$-electrons are precessed (transit from one valley into another) in the same way near the Dirac point because the hexagonal symmetry levels transitions between the levels with different projections j=±3/2,±1/2 of the $\pi (p_{z})$-electron orbital momentum $J_{p_{z}}$ due to the smallness of the spin-orbital coupling (SOC) at momenta $q(q^{\prime })\to 0$, $\vec{q}=\vec{p}-\vec{K}$ ($\vec{q}\;{\;}^{\prime }=\vec{p}\;{\;}^{\prime }-{\vec{K}}^{\prime }$). The precessing of the $\pi (p_{z})$-electron must proliferate the vortices (antivortices). When violating the hexagonal symmetry, the large SOC at the high momenta $q(q^{\prime })$ lifts the degeneration of states over the projections *j* that appear as four topological vortex defects (four antivortices) in a T-shape configuration. An atomic chain with two topological defects at the ends implements a pseudo-Majorana particle [66,67]. The T-shape configuration of four vortex defects (four antivortices) is three pseudo-Majorana quasiparticles differing in the combinations of the vortical subreplicas that form them. The number of the pseudo-Majorana modes coincides with the number N=3 of the gauge degrees of freedom of the graphene model and, accordingly, all three Majorana modes differ in flavor. This signifies that the pair of vortical and antivortical subreplicas possesses one of three flavors.

One of the two eigenvalues of the Majorana mass term entering the Hamiltonian of the pseudo-Majorana fermion turns out to be zero. Therefore, one of the pseudo-Majorana particles is composed of two chiral vortex defects, the second one is composed of two nonchiral vortices, and only one vortex is chiral for the third pseudo-Majorana mode. Since the flavor is associated with the chirality, let us call the pseudo-Majorana differently flavored modes the chiral, semichiral, and nonchiral pseudo-Majorana particles $V_{ch},V_{sc},V_{nc}$.

Thus, the pseudo-Majorana fermion graphene model is a topological semimetal. Resulting in the eight subreplicas of the graphene bands, the SOC is capable of competing with the hexagonal symmetry at large energies in the flat bands only. When the pseudo-cubic symmetry holds, the electron-hole symmetry for every graphene band is broken separately, and, correspondingly, the bound vortical and antivortical pseudo-Majorana fermions, forming electrons and holes, which are deconfined by large SOC. These free deconfined pseudo-Majorana particles exist in a very narrow energy range because they reside in the flat area of the graphene bands. Since the velocity $v_{F}$ of the free Majorana configurations tends to zero, the pseudo-Majorana fermions are very heavy.

The gamma quantum resides in the order of $L_{b}/c∼10$ attoseconds in the carbon nanotube bilayer with a thickness, $L_{b}$, of the order of 2 nm. Elastic collisions between the gamma rays and bilayer atoms with a momentum transfer to the electronic graphene subsystem occur very rarely during this time. In this situation, an angular-momentum transfer is most likely, as the gamma radiation quantum is a circularly polarized electromagnetic wave and, correspondingly, its rotating electric field is able to twist the graphene electron density during this time, starting from the creation of the gamma-ray photons in the IRS. In this case, the interaction occurs in the time the gamma quantum resides in the collimator and this time interval is equal to L/c∼1 picosecond. The pseudo-Majorana vortex quasiparticle excitation occurs as a result of the resonant energy pumping to the vortex when the frequencies of the rotation of the electric vector and vortex arms coincide. In accordance with the theoretically predicted deconfinement, the energy of a flavorless vortex comprising the three differently flavored Majorana quasiparticles is equal to three times the energy of the chiral Majorana excitation. The electron density shifts under the pressure of the light wave and, correspondingly, the differently flavored Majorana pairs of vortices start moving. Due to the fact that the negatively charged vortex pulls the positively charged core of the carbon atom into a whirlpool swirl, the bilayer is not ionized.

The model of the interactions of the photons and the electron density by the Compton mechanism is presented in Figure 2b.

### 4.2. Avalanche Binding of Pseudo-Majorana Fermions

The rotating electric field of the circularly polarized gamma radiation quanta twists the electron density around the vortex cores located at the sites of the hexagonal lattice. The high-energy gamma radiation quantum is able to excite a huge number of low-energy electrons in the flat regions of the graphene band structure. The flat electronic bands, $E_{flat}\left(q_{flat}\right)$, of the pseudo-Majorana graphene model present a divergent density of states because there are van Hove singularities in the density of the fermionic state. Since all these states possess the same energy, $E_{flat}$, and, correspondingly, the same momentum, $q_{flat}$, the fermionic states move as a whole and exist as high-energy Majorana one-particle excitations.

Using the calculation results presented in Figure 1 and Figure 2c, one obtains $q_{flat}=K\left(K^{\prime }\right)$ because the vortex “leg” resides in the valley $K(K^{\prime })$ of the graphene Brillouin zone. Since the SOC lifts the eightfold degeneracy at energies of the order of $E_{flat}$, the homotopy group $Z_{12}$ is deformed in the cyclic group $Z_{8}$ and, as a result, the symmetry of the electron subsystem becomes pseudo-cubic.

Let us estimate the current of the topologically nontrivial graphene charge carriers and make a comparison with a current for the pseudo-Dirac fermion graphene model. To do this, let us use the Heisenberg uncertainty principle $\Delta x_{i}\Delta p_{i}\geq \hbar /2$ [68] to find the density of the charge carriers

(9) $$N\leq \frac{4}{\hbar^{2}}\int dp_{x}\;dp_{y}.$$

Here, $\Delta x_{i},\Delta p_{i}$, i=x,y are the deviations of position $x_{i}$ and momentum $p_{i}$ of the graphene charge carrier. We obtain the limits of integration over $\vec{p}$ in the expression for *N* using the following energy condition $\hbar^{2}\omega^{2}=v_{F}^{2}\left(p\right)p^{2}$, which is imposed on a cyclic frequency ω of the graphene charge carrier [69]. Here, $p=|\vec{p}|$. Then, the density, $N_{D}$, of low-energy graphene charge carriers near the Dirac valley $\vec{K}\left({\vec{K}}^{\prime }\right)$, where the energy condition is the linear energy dispersion $\epsilon \equiv \hbar \omega =\pm pv_{F},v_{F}=10^{6}$ m/c, is

(10) $$N_{D}=\frac{4}{\hbar^{2}}\int_{\hbar |\vec{K}|}^{\hbar (|\vec{K}|+\Delta q)}pdp\int_{0}^{2\pi }d\phi ,\;q\ll 1.$$

Let us choose the energy limits from 0 to 0.02 eV in Expression (10) because the pseudo-Dirac model is verified by comparing the results of the simulation and experiment in this energy region (see [70,71] and references therein). Then, being in perfect agreement with the maximal charge density experimentally observed in the low-energy electrophysical experiments [50], the theoretically predicted value of $N_{D}$ is of the order of

(11) $$N_{D}=\frac{8\pi }{{\left(\hbar v_{F}\right)}^{2}}\int_{0}^{0.02\;eV}\epsilon d\epsilon \approx 2.0\times 10^{13}\;{\mathrm{cm}}^{-2}.$$

The Majorana vortex moves as a whole because it resides in the graphene valley only with the wave number $K=|\vec{K}\left|=\right|{\vec{K}}^{\prime }|$. Let us find its energy. The Majorana vortex is a precessing electron $\pi (p_{z})$ in which the total angular momentum changes its direction on an angle of up to 90${}^{\circ }$. This means that the electron is approaching the valley $K^{\prime }$ from *K* because when bypassing the lattice site, the topologically nontrivial graphene charge carriers with the cyclic group $Z_{12}$ acquire a phase equal to 30${}^{\circ }$. A pseudo-Majorana mode frequency, ω, is determined by the energy condition

(12) $$\omega^{2}={\left.v_{F}^{2}\left(p\right)\right|}_{\vec{p}\to {\vec{K}}^{\prime }}p^{2}.$$

After changing p→p/ℏ, the density, $N_{M}$, of the high-energy pseudo-Majorana fermions is equal to the following expression:

(13) $$N_{M}=4Kv_{F}\left({\vec{K}}^{\prime }\right)\int_{p=p_{M}}\frac{p}{Kv_{F}\left(\vec{p}\right)}dp\int_{0}^{2\pi }d\phi ,\;v_{F}\left(\vec{p}\right)\to v_{F}\left({\vec{K}}^{\prime }\right).$$

Here, ${\vec{p}}_{M}$ is the momentum of the Majorana particle, angular frequency ω, and wave vector $\vec{p}$ , which form a 3-vector. The property

(14) $$\delta \left(f(k)\right)=\frac{1}{{\left.\frac{df}{d\omega }\left(\bar{k}\right)\right|}_{f(\bar{k})=0}}\delta \left(\omega -Kv_{F}\left(p\right)\right)$$

is attributed to the Dirac δ-function depending on the following function:

(15) $$f=k^{2}=\omega^{2}-v_{F}^{2}\left(p\right)p^{2}.$$

Here, *k* is the 3-vector with the following components: $k=(\omega ,v_{F}\left(p\right)\vec{p})$. Then, using Property (14), one obtains

(16) $$\int_{p=p_{M}}\frac{p}{Kv_{F}\left(\vec{p}\right)}dp=\int_{p=p_{M}}\int \delta \left(\omega -Kv_{F}\left(p\right)\right)\frac{2p}{{\left.\left|\frac{\partial f}{\partial \omega }\left(\omega \right)\right|\right|}_{\omega =Kv_{F}\left(p\right)}}d\omega \;dp,\;v_{F}\left(\vec{p}\right)\to v_{F}\left({\vec{K}}^{\prime }\right).$$

Taking into account that $K\left(K^{\prime }\right)∼0.4\times 10^{10}$ m${}^{-1}$, the substitution of Equation (16) into Equation (13) gives the following estimate of the Majorana state density:

(17) $$\begin{array}{c} N_{M}∼16\pi Kv_{F}\left({\vec{K}}^{\prime }\right)\int_{0}^{Kv_{F}\left({\vec{K}}^{\prime }\right)}d\omega \int pdp\delta \left(\omega^{2}-v_{F}^{2}\left({\vec{K}}^{\prime }\right)p^{2}\right)=\frac{8\pi K}{v_{F}\left({\vec{K}}^{\prime }\right)}\int_{0}^{Kv_{F}\left({\vec{K}}^{\prime }\right)}d\omega \\ \approx 4\times 10^{16}\;{\mathrm{cm}}^{-2}. \end{array}$$

The theoretically predicted value (17) of the state density corresponds to a current density of more than $10^{12}$ A cm${}^{-2}$, which is necessary to deliver a sufficient number of electrons neutralizing ions at femtosecond scales [14].

The energy losses of $\pi (p_{z})$-electrons in the Coulomb scattering on carbon atoms render the hexagonally symmetric electron density energetically favorable because of a weakness of the SOC in the system possessing hexagonal symmetry and the $Z_{12}$ homotopy group in comparison with the SOC for the pseudo-cubic symmetry and the $Z_{8}$ group. Correspondingly, the hexagonal symmetry degenerates the graphene Majorana states eightfold, and the confinement binds the Majorana fermions. Having been collected by the hexagonal lattice in the same site as the four vortical (antivortical) electron (hole) subreplicas of the graphene valence (conduction) band and the four antivortical (vortical) hole (electron) subreplicas of the graphene conduction (valence) band, the six Majorana vortical (antivortical) fermions of different flavors produce flavorless electron-hole vortical (antivortical) configurations. The deconfined pseudo-Majorana fermions act as quasiparticles jumping between the electron and hole valleys of the graphene Brillouin zone, and, correspondingly, an electrical charge cannot be rendered to these quasiparticles. When confining the pseudo-Majorana fermions in the electron and hole valleys, the hexagonal symmetry renders the negative and positive electrical charges to the Dirac massless electron and hole configurations. When the pseudo-Majorana fermion “jumps” between the valleys $\vec{K}$ and ${\vec{K}}^{\prime }$, the multipliers $\exp(\mp \left(\vec{K}+\vec{q}\right)\cdot \vec{r})$ and $\exp(\pm \left({\vec{K}}^{\prime }+\vec{q}\right)\cdot \vec{r})$ entering the corresponding components of its bispinor wave function acquire an added phase equal to $\frac{\pi }{2}$ because the vector $\vec{K}\left({\vec{K}}^{\prime }\right)$ is rotated by the angle equal to $\frac{\pi }{3}$ by virtue of the hexagonal symmetry of the Brillouin zone, and the radius-vector $\vec{r}$ of the charge carriers is rotated by the Zak phase equal to $\frac{\pi }{6}$ after bypassing the topological defect, which is the vortex core residing in the Dirac point $\vec{K}\left({\vec{K}}^{\prime }\right)$ (see Figure 2c). Correspondingly, the quasi-relativistic electron and hole ohmic contributions to the total current are orthogonal to each other [71]. Hence, the non-zero resulting ohmic current when flowing in the direction of the applied electric field, $\vec{E}$, may neutralize the radiation-induced electrically charged defects because by non-overlapping each other, the orthogonal electron and hole currents cannot destroy each other. Each vortex band subreplica exists in a pair with its own antisubreplica of the opposite vorticity. This means that the vortices are created in pairs with a total zero topological charge. The law of the topological charge conservation is satisfied for eightfold-degenerated vortices and is revealed through the dichroism of the graphene band (see Figure 1 and Figure 2c). After colliding with the graphene lattice, the pseudo-Majorana vortical configurations acquire different energies and, as a consequence, a mismatch in the energy locations of the different branches of the eight vortices from the flat bands arises from the disintegration of the structure of the free Majorana state into separate “vortex-branch” configurations. Since the extent of the disintegration becomes avalanche-like because of the appearance of the hexagonal symmetry, the confinement decay channel allowed in the disintegrated Majorana fermion, $V_{i}$, gives plenty of Dirac electron-hole pairs in the following manner:

(18) $$V_{i}\to n_{i}^{e}e^{-}+n_{i}^{h}h^{+},\;n_{i}^{e},n_{i}^{h}\gg 1;\;i=ch,sc,nc.$$

Here, the total number of electrons coincides with the total number of holes $\sum_{i}\left(n_{i}^{e}-n_{i}^{h}\right)=0$.

Thus, an electron-hole pair avalanche is produced due to the confinement of the Majorana fermions in the flavorless Dirac configurations. The prediction of the low-energy electron avalanche explains the appearance of the large number of low-energy electrons (0–10-eV electrons) that are experimentally observed in the irradiated graphene samples [22]. The vortical electron density swirls not only around the carbon atom but also around the impurity ion falling on the graphene plane from an irradiating ion beam. The entrained ion projectile is neutralized by the avalanche of Dirac states of the bound (confined) pseudo-Majorana fermions. The prediction explains the neutralization of the beam in graphene [14].

We justify the pseudo-Majorana nature of graphene fermions through our experimental results.

## 5. Analysis of Radiation Spectra

### 5.1. Escaping Absorber-Scattered Gamma Rays

Hereafter, experimental evidence is presented to show that pseudo-Majorana vortical fermions exist on the defects induced by the gamma-ray irradiation of the graphene plane.

**Figure 5:**
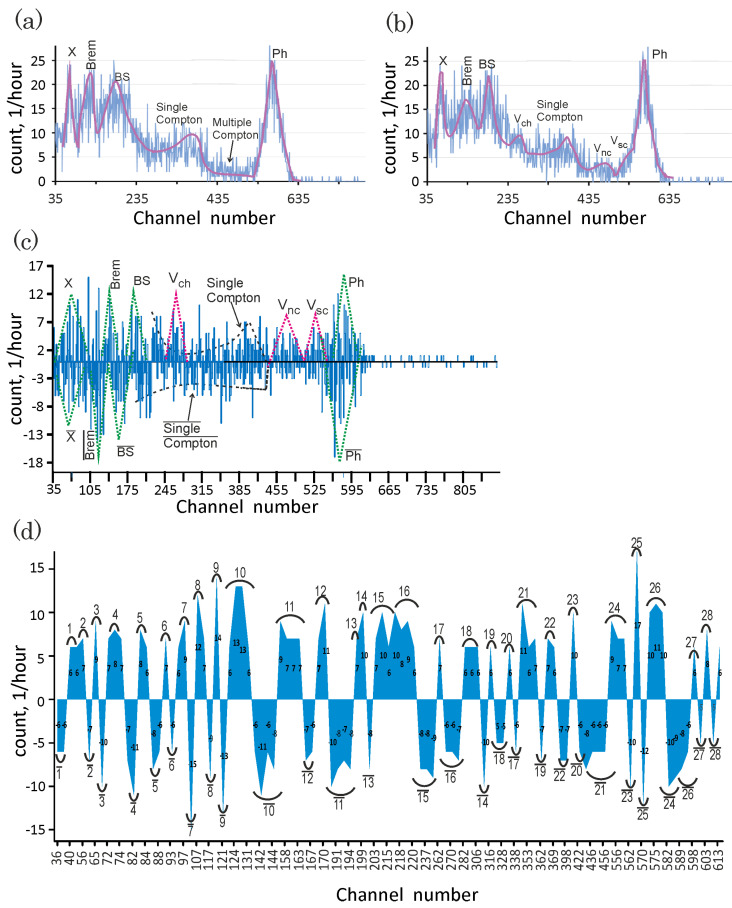
(**a**) The pulse height spectrum, $R_{Cs}$, of the secondary electrons induced by the ${}^{137}$Cs gamma quanta scattering on the crystal detector and (**b**) the pulse height spectrum, $R_{CsG}$, of the secondary electrons collected following the impact of the ${}^{137}$Cs gamma quanta on the bilayer target; the radiation background has been subtracted from the original distributions. (**c**) The difference, ΔR, between the distributions of $R_{CsG}$ and $R_{Cs}$: $\Delta R=R_{CsG}$−$R_{Cs}$; the inverted peaks (antipeaks) related to the spectrum $R_{Cs}$ are marked by an overline. (**d**) Histogram of the bands and antibands formed by the channels of the difference spectrum inverted with respect to the spectrum in figure (**c**) for which the modules exceed 5 pulses/hour; the number of events for each channel of the bands and antibands is given before and after the placement of the absorber into the collimator, respectively.

Let us analyze the carbon nanotube bilayer effects on the incoming ${}^{137}$Cs gamma-quanta beam. A comparison of Figure 2d–e and Figure 5a–c and Table 1 shows the following features of the radiation spectra of the aligned carbon nanotube assemblies decorated by the organometallic compound under investigation. Let us denote by $N_{Cs}^{ph}$ and $N_{Cs/CNT}^{ph}$ the numbers of the photopeak events that were recorded before and after the placement of the bilayer sample into the collimator, respectively. $N_{Cs}^{ph}$ and $N_{Cs/CNT}^{ph}$ are equivalent to the following expressions: $N_{Cs}^{ph}=Ah_{1/2}^{Cs}=833$ and $N_{Cs/CNT}^{ph}=Ah_{1/2}^{Cs/CNT}=625$, respectively. Here, *A*, A≈25 is the photopeak amplitude; $h_{1/2}^{i}$, i=Cs,Cs/CNT is the half peak width. Accordingly, the placement of the absorber into the collimator causes a decrease in the number of events recorded in the photopeak by 208±2 pulses per hour. Subtracting the total radiation background $N_{bg}$ from the experimental data, one finds the total numbers $N_{Cs}$ and $N_{Cs/CNT}$ of the events in the detector with and without the target in the ${}^{137}$Cs gamma-quanta beam are equal to 5191±16 and 5111±15, respectively. $N_{Cs}$ and $N_{Cs/CNT}$ are connected within the standard phenomenology of gamma-ray attenuation by the relationship

(19) $$N_{Cs/CNT}=N_{Cs}\exp\left(-2\mu d_{G}\right)$$

where μ is the probability of the interaction of the photons per unit length of the path in the graphene monolayers and $d_{G}$ is the thickness of the atomically thin layer $(∼10^{-10}$ m); factor 2 takes into account approximately two layers of carbon atoms interacting with the radiation. The assessment of μ from this formula with the data given above yields

(20) $$\mu ∼0.8\times 10^{7}m^{-1}.$$

If one interprets μ as a macroscopic cross-section of 3D objects, the result (20) would provide the cross-section estimate of the order of $10^{6}$ barn. This means that the conventional concept of the macroscopic cross-section is not applicable in this case. Nevertheless, the sufficient drop in the counts observed in the detector after the radiation passed through the bilayer film warrants further examination of the topologically nontrivial aspects inherent in the scattering of projectiles on entire carbon atoms in graphene.

After placing the electromagnetic radiation bilayer absorber into the collimator, three additional peaks appear in the ${}^{137}$Cs radiation spectrum of the secondary electrons along with the photopeak, single Compton continuum, backscatter peak, characteristic X-ray peak, and bremsstrahlung [49]. The three new peaks with the maxima approximately in the 270th, 475th, and 535th channels (compare Figure 5a,b) indicate that the gamma quanta escape from the detector by creating vortex pairs of electron density, which are neutral pseudo-Majorana fermions in graphene. A comparison of the $R_{CsG}$ and $R_{Cs}$ spectra indicates a narrowing of the ${}^{137}$Cs radiation peaks. The shape of the single Compton continuum of the ${}^{137}$Cs radiation spectrum becomes steeper after placing the bilayer sample into the collimator. The peak “Brem” decreases by a factor of two when the photons from the bremsstrahlung process interact with the rolled-up graphene plane.

The spectra $R_{CsG}$ and $R_{Cs}$ recorded with and without the bilayer absorber do not cancel out each other. The difference in the response functions is presented in Figure 5c. The difference spectra include all characteristic peaks of both response functions in the form of the peaks recorded by the crystal detector–absorber system and the inverted peaks (antipeaks) recorded by the crystal detector. The positions of the peak maxima and antipeak minima in the difference spectra are shown in Table 1. The peaks in $R_{CsG}$ are shifted from the locations of the peaks in the primary response function. As Figure 5c shows, after colliding with the carbon nanotube bilayer, the gamma quanta are redistributed in all channels, except for the multiple Compton scattering events. We intend to interpret this as the multiple Compton scattering leading to multiple changes in the direction of movement of the incident photons occurring only in the detector crystal. Since the energy deposition remains for the multiple-scattering channel only, the increase in the deposited energy from the photoelectric absorption, characteristic X-ray photons, bremsstrahlung, and backscattering occurs due to the scattering of the IRS beam on the bilayer only.

When comparing the single Compton continuums in the radiation spectra for the detector with and without the sample, we conclude that the growth in the number of photons scattered at the scattering angle θ=π is observed in the presence of the bilayer sample (see Figure 5c). In accordance with the Klein–Nishina formula [72], the number of photons scattered at θ=π grows by decreasing their energy [73]. Therefore, the change in the shape of the single Compton continuum from flat to bent indicates a decrease in the flux of the ${}^{137}$Cs gamma quanta due to the energy deposition in the sample.

The semimetal graphene hosts bound electrons (electrons in steady states) and, correspondingly, the uncharged photons cannot influence the graphene surface through the Coulomb force. The effects of the gamma-ray irradiation of the bilayer reveal that there is a Compton mechanism of the interaction of gamma rays with graphene. This mechanism does not involve free electrons, but the free electrons appear after the radiation-induced creation of vortical defects in graphene.

### 5.2. CNT-Enhanced Deposition of Energy in Detector

Now, let us estimate the variation in the energy deposition in the channels after the installation of the sample in the collimator. To achieve this, let us select groups of channels so that six and more credible events are recorded in each channel of the groups (see Figure 5d). Let us call these channel groups bands. The bands from $R_{Cs}$ are shifted after the sample is installed in the collimator. Let us characterize the scattering graphene centers by analyzing the band shifts. A redistributed band is defined as a band in the radiation spectrum, $R_{CsG}$, of the IRS shielded by the sample, with the number of events close to the number of events for the primary band. The locations of the bands will be defined by the averaged values of the numbers of channels belonging to these bands. These primary and redistributed averaged channel numbers, as well as the values and directions of their shifts with respect to each other, are represented in Table 2.

According to the data shown in Table 2, after interacting with the graphene CNT walls, the gamma quanta scattered in the detector crystal into channels corresponding to the bands, which are labeled with numbers from 14 to 23, are redistributed into the bands formed by channels with higher numbers. The primary band, for example, “16”, which is in the region of the single Compton continuum, is transferred into band “$\bar{16}$” so that the averaged channel number is shifted from 218 to 269. The primary bands, whose averaged numbers are 198, 333, and 404, are shifted to the three channel areas near the 311th, 421st, and 559th redistributed channels. This means that after interacting with the carbon nanotube bilayer, the photons originally recorded in the 14th, 20th, and 23rd bands “are trapped” in the channels belonging to bands “$\bar{14}$”, “$\bar{20}$”, and “$\bar{23}$” and shifted with respect to the primary ones on (+113), (+88), and (+154), respectively (see Table 2). The locations of bands “$\bar{14}$”, “$\bar{20}$”, and “$\bar{23}$” correlate with the positions of the sample radiation peaks, whose maxima are near the 270th, 475th, and 535th channels. The huge radiation band shifts with respect to the primary bands demonstrate the scattering of photons on the super-heavy fermions in the graphene plane.

Thus, the positive shifts mean that the increased number of photons with higher energies are registered by the detector. Since other energy loss peaks of characteristic X-ray photons do not appear in the radiation spectrum, the collisions with the gamma ray do not lead to the ionization of the bilayer.

## 6. Discussion

### 6.1. Interaction of 661.7-keV Gamma Rays with Graphene Sheet

In this section, we discuss the proposed mechanism of the interaction of the rolled-up graphene with the gamma radiation, which is particularly relevant in light of the experimental facts reported above.

When swirling an electron density in the graphene plane by the rotating electric field, the gamma quantum creates a feather of a vortex, whose core resides in the Dirac point. The characteristics of these topologically nontrivial radiation-induced defects are non-Abelian and the pseudo-Majorana quasiparticle fermion excitations reside on the defects (see Section 4). This means that a fraction of the photon energy is absorbed in the bilayer to produce the three differently flavored vortical Majorana particles. This is recorded as three additional peaks, $V_{ch}$, $V_{sc}$, and $V_{nc}$, in the radiation spectrum of the secondary electrons of the detector crystal–bilayer system in comparison to the gamma-radiation spectrum recorded in the absence of the bilayer sample (see Figure 2d–e). The existence of these peaks confirms the above theoretical prediction that gamma rays excite high-energy differently flavored quasiparticle states in the graphene plane.

The graphene pseudo-Majorana fermions are massless at the Dirac point $K(K^{\prime })$ of the graphene Brillouin zone. However, outside the $K,K^{\prime }$ valleys in the conduction and valence bands, either one vortex (antivortex) from the pseudo-Majorana pair remains massless and the other acquires a pseudo-Majorana mass, or the two vortices are massless or both the vortices acquire the mass [65].

The massless pseudo-Majorana fermions move collisionless in the graphene plane by virtue of the laws of conservation of helicity and topological charge. Correspondingly, they live until the pairs of bound pseudo-Majorana fermions with zero topological charge are formed. Since the lifetime τ of the free Majorana fermions is much longer than the detection time, the energy, $E_{V_{ch}}$, released in the detector becomes less than the ${}^{137}$Cs gamma quantum energy, $E_{0}$, on the energy $E_{ch}$ of the chiral pseudo-Majorana fermion $V_{ch}$ : $E_{ch}=E_{0}-E_{V_{ch}}$. The peak labeled as “$V_{ch}$” in Figure 2e is at the energy $E_{V_{ch}}$ and, correspondingly, the energy of the one massless pseudo-Majorana mode in the graphene plane is of the order of 173 keV.

The nonchiral vortices with the non-zero pseudo-Majorana mass stop and then remain in a resting state until the disintegration proceeds. The de-excitation of the semichiral pseudo-Majorana fermion $V_{sc}$ is accompanied by the release of a characteristic photon, $\gamma_{v}$, and the energy of the disintegrated Majorana fermion is deposited in the detector as a result of the transition to the separate branch states. Since the event happens in the graphene plane, the characteristic X-ray $\gamma_{v}$ cannot be absorbed into the detector after scattering in the graphene lattice and, correspondingly, the energy deposited in the detector decreases by an amount equal to $E_{b}$. Correspondingly, the peak $V_{sc}$ in the radiation spectrum for the IRS with the absorber is at the energy $E_{V_{sc}}=E_{0}-E_{b}$ with $E_{b}∼20$ keV (see Figure 2e).

The right and left twisted vortices entering the nonchiral pseudo-Majorana fermion, $V_{nc}$, possess non-zero Majorana masses. Therefore, the pseudo-Majorana fermion, $V_{nc}$, de-excites by emitting a characteristic photon $\gamma_{2v}$ and stops. The fermion $V_{nc}$ being in the resting state disintegrates by depositing the energy in the detector through the confinement decay channel. This means that the energy deposited in the detector decreases by an amount equal to $2E_{b}∼40$ keV and the peak $V_{nc}$ in the radiation spectrum for the IRS with the absorber is at the energy $E_{V_{nc}}=E_{0}-2E_{b}$ (see Figure 2e).

As a result, based on the experimental data, one can estimate the energy, $E_{dec}$, needed to deconfine the bound pseudo-Majorana fermions. $E_{dec}$ is equal to three times the energy of the chiral Majorana mode, $E_{dec}=3E_{ch}=519$ keV, and the energy of the disintegrated Majorana fermion, $E_{dis}$, is equal to $E_{dis}=E_{dec}-3E_{b}=459$ keV.

Placed outside the sites of the pseudo-cubic lattice, the Majorana vortices in flavor pull carbon atoms in a circular motion and, correspondingly, three differently flavored ordered sets of circular apertures appear as the electron density, and carbon atoms are absent in the sites of the pseudo-cubic lattice. The incident gamma rays undergo Fraunhofer diffraction on these voids. The presence of the three radiation spectrum bands undergoing the giant shifts ({(+88),(+113),(+154)}) toward larger numbers of detector channels (see Table 2) is an experimental confirmation of the theoretically predicted existence of the Fraunhofer diffraction at the empty sites of the pseudo-cubic vortex lattice.

Thus, it is legitimate to assume that graphene radiation-induced defects of three types exist for a long enough time to be depolarized, interacting with each other and losing energy in collisions with the graphene lattice. The gamma rays are bent through the empty cores as apertures into the region of the geometrical shadow of the Majorana fermions. As a result, the probability of gamma-ray scattering in the detector crystal with the subsequent additional production of primary photoelectrons will increase because there are limited regions around the diffracting vortex cores where photons are more likely to originate from.

The presence of the bands, whose displacements are weaker, is discussed in the next paragraphs.

### 6.2. Klein Tunneling through Electrostatic Barrier Generated by Scattering on Vortical Radiation-Induced Defects in the Rolled-Up Graphene Sheets

In this section, we present experimental evidence to show that the Majorana modes can also be revealed through scattering on resonance states created at oblique incidence on an electrostatic barrier as a result of Klein tunneling. Klein-type tunneling is featured only in the graphene hexagonal lattice hosting fermions, whose characteristics are non-Abelian [55].

#### 6.2.1. Doping Effect of Pseudo-Majorana Mode Creation in Laser Fields

The vortices of electron density in the graphene plane are also created under the action of the rotating electrical field of the laser radiation. In this case, the displacement of the vortex core is very small, but the electron density swirling around the carbon nuclei still causes them to be pulled along like a ”wall” in the vortex funnel. Since the empty vortex cores play the role of vacancies, the graphene plane is effectively doped by holes and, correspondingly, the Dirac point is positively charged. The graphene-free charge carriers swing in the electric field of the organometallic five-monolayer LB film in resonance with its molecular vibrational modes, excited by the electric field of the laser radiation. These plasma-forced oscillations reproduce the high-intensity Fe(II)DTP Raman spectrum shown in red in Figure 3b. The CNT enhancement of light scattering was also observed in [74,75].

Thus, the mechanism of interaction of laser radiation and graphene stems from the existence of the pseudo-Majorana mode.

#### 6.2.2. Scattering of Charge Carriers on Electron Beam Induces Defects of Pseudo-Majorana Type

The photons are not electrically charged, and, correspondingly, do not produce charge carrier currents. However, these currents can be created by an electron beam that pushes electrons out of the lattice sites, electrically polarizing the graphene crystal cell. The analysis of the electron structure of the graphene surface using TEM demonstrates that the electric field of the 100 keV electron beam oblique to the aligned CNT assemblies “negatively dopes” the graphene plane. These “negatively doped patches” are visualized as an “electron-dense layer”. The apparent CNT diameter (1.9 nm) increases 2.5 times in comparison to the size of the nanotube cross-section (0.761 nm) due to this “doping” (see Figure 4b,c). On the contrary, the transversely incident electron beam does not dope the bilayer and the CNT assemblies (see Figure 4b,c). The electron beam is a moving electron bunch described by a wave packet. When this electron bunch collides with a graphene patch, this patch is “positively doped” because the vortex funnels are excited. Meanwhile, the vortex cores of the Majorana fermions are not filled by any charge density. Graphene electrons that are driven by the electric field of the negative bunch incident obliquely are scattered on these positively doped graphene patches and form resonances known as electrostatically confined quantum dots, which arise as a result of Klein tunneling. The Fraunhofer diffraction of the 100-keV electron beam occurs on the graphene quantum dots because they are voids. The diffraction patterns, which look like the “electron-dense” layers shown in the TEM images in Figure 4b,c, are a result of the Fraunhofer diffraction.

### 6.3. γ-Ray Diffraction on Radiation-Induced Vacancy Clusters in Single-Layer Graphene

The high-energy vortex of the pseudo-Majorana fermion flow in flavor is capable of carrying electrically neutral carbon atoms that are pulled into the vortex funnels away from their locations in the hexagonal lattice. Since the antivortices entering these pseudo-Majorana fermions remain in the lattice sites where the atoms previously resided, a cluster of vacancies that are electrically neutral but different in flavor is formed. Meanwhile, the flows of the vortices belonging to these Majorana fermions carry away electrically neutral carbon atoms (see the model shown in Figure 2b). The radiation-induced defect of the type of electrically neutral vacancy, $V_{g}^{0}$, in flavor is stable, as annihilation between it and flavorless negatively charged graphene states is forbidden by the law of conservation of the topological charge. The radiation-induced vacancies in flavor differ also in the direction of vorticity (right-handed or left-handed). This means that there are at least six types of electrically neutral vacancies. The spaces of the clusters of differently flavored vacancies are not filled with anything; hence, the backscatter and bremsstrahlung 1 nm radiations are diffracted by these voids by slits. The pulse heights for the backscatter and bremsstrahlung channels might increase because the bending of the backscatter and bremsstrahlung photons on the radiation-induced vacancies in flavor facilitates the energy deposition to the detector. Verifying the existence of the six types of vacancies in flavor in the experimental data shown in Table 2 indicates small and very small shifts in the radiation bands. These small and very small shifts are arranged in the following two sets consisting of three two-element groups: (1) ((33,40);(48,51);(77,84)), and (2) ((12,18);(24,28);(30,33)). This signifies that the backscatter and bremsstrahlung photons are diffracted by the semichiral, nonchiral, and chiral vacancies. The first shift set ((33,40);(48,51);(77,84)) appears due to scattering of the backscatter photons in the bilayer sample and the second one ((12,18);(24,28);(30,33)) appears due to the scattering of bremsstrahlung photons in the sample because these additional contributions to the energy deposition into the detector must be greater for higher-energy backscatter photons than for bremsstrahlung ones. The existence of the sets indicates the theoretically predicted electrically neutral vacancies in flavor.

Thus, by changing the direction of the wave vector, the diffraction effectively increases the number of trapped photons. The longer-wave bremsstrahlung photons that are deflected at very large angles are capable of going around an obstacle of such sizes as the vacancy in flavor. The intensity of the “Brem” peak decreases by a factor of two due to the interference of the bremsstrahlung photons. The flavored vortices carrying out the electrically neutral carbon atoms are in a quasi-steady state by virtue of the law of conservation of the topological charge. The topological charge of the radiation-induced defect of the “vacancy in flavor” can be neutralized only by the vortex bringing the carried-away topological charge and knock-on carbon atom to the vacancy; after that, the radiation damage disappears.

This discovery, together with the contraction of the Compton contribution and the possibility of reducing the sizes for both the crystal and radiation shield, allows for the development of inexpensive detectors with higher collection efficiency. The presence of the electron-hole avalanche impedes the creation of defects in the detector crystal at exposure, and, correspondingly, can facilitate the increase in the operating lifetime of the detector crystal. Our findings open up prospects for a dramatic improvement in the sensitivity of modern detectors.

## 7. Conclusions

The experimental evidence shows that very high energetic vortex electron density structures can reside in monolayer graphene. A very large number of the vortex structure branches hosted by the flattenings of graphene bands behave as a whole. Pairs of topologically nontrivial vortical and antivortical defects are created in rolled-up graphene planes irradiated by the 661.7-keV gamma quanta. The high-energy graphene vortex–antivortex pairs are pseudo-Majorana fermions.

We provide the mechanism responsible for graphene radiation hardness, according to which gamma rays can escape from the detector crystal–CNT bilayer system due to the production of irradiation defect pairs. When creating neutral radiation-induced vacancies $V_{g}^{0}$, the vortical pseudo-Majorana fermions confine the knock-on neutral carbon atoms C${}^{0}$ in the graphene plane. The graphene radiation hardness mechanism adequately explains the features of the radiation spectra of ${}^{137}$Cs that are shielded by the aligned carbon nanotube bilayers.

We theoretically predict and experimentally demonstrate that the Fraunhofer diffraction of the gamma quanta on the vortex of the pseudo-cubic crystal lattice with non-occupied sites and the Fraunhofer diffraction of the backscatter and bremsstrahlung γ-rays on the neutral vacancies, $V_{g}^{0}$, in flavor provides the energy excess. These diffraction phenomena effectively increase the detector size due to the unprecedented high performance in trapping the gamma-ray photons.

Finally, the incorporation of the suspended metallic carbon nanotube assemblies into the collimator greatly increases the beam-energy deposition in the crystal detector and radiation shield due to the electron-hole avalanche that occurs in the disintegrated Majorana fermion’s confinement decay channel. Through our research, we have found that the detection of ionizing radiation with very low levels that are below the detection limit of conventional detectors can be achieved via the measurement of the conductivity change of thin films, which allows us to determine the presence of radiation.

## Figures and Tables

**Table 1:** Maximal channel numbers for the characteristic peaks “*X*”, “Brem”, “BS”, “Ph”, “$V_{i}$”, i=ch,sc,nc and the region of the single Compton continuum in the response functions, $R_{Cs}$ and $R_{CsG}$, recorded without and with the CNT bilayer, respectively.

Name of Characteristic Peak	$R_{\mathrm{Cs}}$-Maximum Number	$R_{\mathrm{CsG}}$- Maximum Number	Assignment
*X*	68	74	Characteristic X-rays from the lead collimator
Brem	124	135	Bremsstrahlung process
BS	179	190	Compton backscattering in materials
			surrounding the detector
Ph	567	572	Photoelectric absorption
$V_{ch}$	–	270	Production of the chiral vortex pair
$V_{sc}$	–	475	Production of the semichiral vortex pair
$V_{nc}$	–	535	Production of the nonchiral vortex pair
Single Compton	210–392	223–386	Single Compton scattering

**Table 2:** Shifts in the ${}^{137}$Cs-radiation spectrum bands in the presence of the bilayer absorber. Averaged numbers of events for the primary and redistributed bands are denoted by $N_{p}$ and $N_{r}$, respectively.

Numbers of Primary and	Averaged Primary-	Averaged Redistributed-	Band
Redistributed ${}^{137}$Cs Bands	Channel Number; $N_{p}$	Channel Number; $N_{r}$	Shift
1 and $\bar{1}$	40; 12	36; 12	−4
2 and $\bar{2}$	56; 7	63; 7	+7
3 and $\bar{3}$	65; 9	67; 10	+2
4 and $\bar{4}$	73; 22	82, 18	+9
5 and $\bar{5}$	85; 14	88; 14	+3
6 and $\bar{6}$	92; 7	93; 6	+1
7 and $\bar{7}$	94; 15	102; 15	+8
8 and $\bar{8}$	107; 19	112; 9	+5
9 and $\bar{9}$	119 ; 14	121; 13	+24
10 and $\bar{10}$	124; 38	142; 31	+18
11 and $\bar{11}$	158; 30	191; 33	+33
12 and $\bar{12}$	169; 18	165; 13	−2
13 and $\bar{13}$	196; 7	201; 8	+5
14 and $\bar{14}$	198; 10	311; 10	+***113***
15 and $\bar{15}$	203; 28	233; 25	+30
16 and $\bar{16}$	218; 28	269; 19	+51
17 and $\bar{17}$	261; 7	338; 6	+77
18 and $\bar{18}$	283; 18	323; 10	+40
19 and $\bar{19}$	313; 6	361; 7	+48
20 and $\bar{20}$	333; 6	421; 6	+***88***
21 and $\bar{21}$	351; 24	446; 26	+84
22 and $\bar{22}$	367; 13	395; 14	+28
23 and $\bar{23}$	404; 10	559; 10	+***154***
24 and $\bar{24}$	548; 23	581; 10	+33
25 and $\bar{25}$	563; 17	567; 12	+4
26 and $\bar{26}$	573; 31	585; 23	+12
27 and $\bar{27}$	597; 6	600; 5	+3
28 and $\bar{28}$	602; 8	603; 5	+1

## Data Availability

Not applicable.
